# Laser Metal Deposition of Rene 80—Microstructure and Solidification Behavior Modelling †

**DOI:** 10.3390/mi15101234

**Published:** 2024-09-30

**Authors:** Krishnanand Srinivasan, Andrey Gumenyuk, Michael Rethmeier

**Affiliations:** 1 Bundesanstalt für Materialforschung und -prüfung (BAM), Unter den Eichen 87, 12205 Berlin, Germany; krishnanandsrinivasan@gmail.com (K.S.); michael.rethmeier@bam.de (M.R.); 2 SHW Brake Systems GmbH, Ludwigstal 25, 78532 Tuttlingen, Germany; 3 Institute of Machine Tools and Factory Management, Technische Universität Berlin, Pascalstraße 8-9, 10587 Berlin, Germany; 4 Fraunhofer Institute for Production Systems and Design Technology, Pascalstraße 8-9, 10587 Berlin, Germany

**Keywords:** laser metal deposition, solidification behavior, analytical model, nickel-based superalloy, additive manufacturing

## Abstract

New developments in nickel-based superalloys and production methods, such as the use of additive manufacturing (AM), can result in innovative designs for turbines. It is crucial to understand how the material behaves during the AM process to advance the industrial use of these techniques. An analytical model based on reaction–diffusion formalism is developed to better explain the solidification behavior of the material during laser metal deposition (LMD). The well-known Scheil–Gulliver theory has some drawbacks, such as the assumption of equilibrium at the solid–liquid interface, which is addressed by this method. The solidified fractions under the Scheil model and the pure equilibrium model are calculated using CALPHAD simulations. A differential scanning calorimeter is used to measure the heat flow during the solid–liquid phase transformation, the result of which is further converted to solidified fractions. The analytical model is compared with all the other models for validation.

## 1. Introduction

Additive manufacturing has gained significant recognition in the energy industry for its ability to produce components, such as gas burners, which offer improved combustion efficiency and reduce the environmental impact of energy production [1]. Traditionally, the manufacture of a gas burner requires the assembly of 13 separate parts and 18 welds. However, in 2017, Siemens successfully produced a gas burner for a gas turbine at a combined cycle power plant in Germany using selective laser melting (SLM) technology [2].

Nickel-based superalloys like Rene 80 are commonly used in high-temperature applications in the energy and aerospace industries due to their high creep resistance, fatigue strength, corrosion resistance, high melting point, and oxidation resistance [3,4]. The Rene 80 alloy consists of a solid solution matrix primarily composed of the γ phase (Ni) and reinforced with solid solution elements like Co and Cr. This is the main phase formed in the course of solidification. The secondary phase is L12 (γ′), which possesses a face-centered cubic (FCC) crystal structure, with varying morphologies based on Al and Ti weight percentages. In the volume ratio, the content of the hardening intermetallic γ′ phase can exceed 50–55%. Also, there can be inclusions, δ phase, and various metal carbides, MC/M6C [5]. The formation of γ″ precipitates is not expected in Rene 80 due to the absence of Nb [6]. The main problem for this alloy in additive processes is the formation of hot cracks, primarily associated with the misfit of the γ and γ′ phases’ lattice [3,7,8,9].

The growing interest in additive production technologies has contributed to the development of the mathematical modeling of physical and metallurgical processes [10,11,12,13,14,15,16] in order to better understand the mechanisms of phase transformation under conditions characteristic of additive technologies and, ultimately, to better predict the microstructure and properties of the final products. Despite the great similarity, the laser cladding process is considered to be more prone to the formation of hot cracks in the manufacture of products from alloys such as Rene than welding because the zone of thermal influence during direct laser deposition is formed layer by layer. This means that the metal can be reheated multiple times, resulting in the material remelting and resolidifying, which can result in the formation of certain defects like liquation cracks. Over the last 10 years, there has been more research on the modelling of the microstructure of heat-resistant nickel alloys, which are typically used in the manufacture of gas turbine parts and parts for rocket and aircraft engines. Much of this research work is devoted to phase transformations and the formation of micro-segregations in the interdendritic space. Different modelling approaches can be used here. Two methods are most commonly used, namely, the stochastic (cellular automat (CA)) method [10,11] and the deterministic (phase field (PF)) method [12], to model dendrite growth during solidification in welding and additive manufacturing. In both cases, one usually considers a two-dimensional mesoscale spatial model, located near the solidification front, which depends on the thermal cycle determined experimentally or by an additional thermal finite element macro-model (FEM). One of the first attempts to describe this phenomenon for the process of direct metal deposition was made by Yin and Felicelli [10]. The authors simulated changes in the cooling rate and temperature gradients for different conditions. It was shown that both the volume of the Laves phase and its distribution change depend on these factors. A finite element bound phase field model has recently been proposed to model similar effects for additive processes [13]. The aim of the analysis was to estimate the effect of the disorientation effect, i.e., the angle between the direction of the temperature gradient and the direction of epitaxial dendrite growth determined by the position of the curing place in the molten pool, on the formation of the micro-segregation zone. Then, in [14], a phase field model was applied for the mesoscale modelling of dendritic solidification. As a result, the authors [14] determined the primary dendritic arm spacing (PDAS) depending on the cooling rate and compared these results with known theoretical models. Similar results were obtained in [15]. Here, the authors considered the influence of the location of dendrites inside the melt pool on the change in their PDAS with the change in the solidification rate and a temperature gradient received from the macro-model. Further, these results were extended by using the CALPHAD calculation of micro-segregation between dendrite branches in the multicomponent system of a real alloy (IN625). In another paper [16], the PF method was used to work with multicomponent alloys using the commercial MICRESS software to predict complex phase transformation during direct laser deposition with the IN718 alloy. Most of the mentioned models consider quasi-equilibrium conditions at the solid–liquid interface, which can hardly be justified for rapid solidification conditions.

The Scheil model, regarded as a ‘non-equilibrium’ model on a macroscale, assumes local equilibrium at the interface (S-L interface) through stepwise equilibrium computations [17]. However, this model fails to explain non-equilibrium effects observed during rapid solidification in AM processes, such as solute trapping, where solutes are trapped within the solid phase [18]. Based on the above review of literature sources, it can be concluded that there is a gap in the understanding of the causes of hot cracking during the additive processes of Ni-based superalloys. The most important task for describing the effect of process parameters on metallurgical micro- and macro-destruction in the material is to develop a microscale model capable of quickly quantitatively analyzing the parameters of the microstructure based on the kinetic approach and taking into account the characteristic conditions of thermal cycling occurring during the deposition process. Hence, we propose an analytical model, presented earlier [19], to describe the non-equilibrium interface kinetics resulting from AM. In the current work, we extend it for application under non-equilibrium conditions during the laser metal deposition process and provide its experimental validation for both slow and rapid solidification.

## 2. Analytical Model

**Figure 1:**
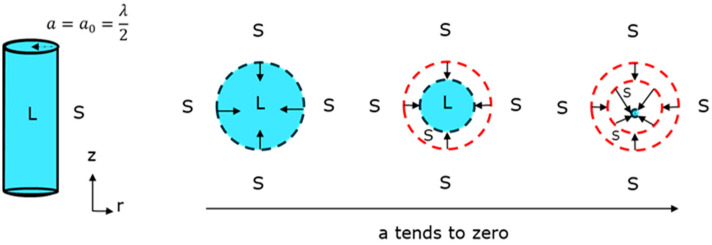
Model domain assumption.

In this study, we present an analytical model which improves the description of the non-equilibrium kinetics at the solidification front and overcomes the issues imposed by the conventional Scheil model. The following fundamental assumptions are adopted in the present model. The liquid ‘a’ of this cylinder is assumed to be half the distance to the dendritic arm spacing (DAS), providing an estimation of the liquid phase geometry. Surrounding this cylindrical liquid phase is an infinite medium of the solid phase. During the solidification process, as the solid phase grows and the liquid phase recedes, the radius of the cylinder gradually decreases. Ideally, when solidification is complete, the cylinder’s radius ‘a’ would reach zero, indicating a fully solidified system (Figure 1). This assumption allows us to define the model domain with a primary focus on the solid–liquid (S-L) interface. It is important to note that our model is currently designed to describe only the primary phase formation, specifically the γ phase originating from the liquid. We have not considered the formation of any secondary phases, such as metallic carbides (MCs) or γ′ phases, in this study.

The concentration profile in the liquid is defined using Fick’s second law of diffusion using Equation (1).

(1) $$\frac{\partial C}{\partial t}=\frac{1}{r}D\frac{\partial }{\partial r}\left(r.\frac{\partial C}{\partial r}\right),$$

The initial solute concentration in the liquid ${C=C}_{0}$ determines the initial condition. At the S-L interface, the boundary condition is established by considering the balance between the diffusion process and the reaction kinetics of phase formation and dissolution, as represented by Equation (2).

(2) $$-D\frac{\partial C}{dr}=K\left(T\right)\;\left(C_{L}\left(T\right)-C\right),$$

where $C_{L}\left(T\right)$ is the equilibrium liquidus concentration at temperature *T*, and $K\left(T\right)$ is the reaction constant. The solution to this boundary value problem (BVP) is a well-known expression derived from the heat exchange between an infinite cylindrical rod surface and the external medium (Carslaw and Jaeger [16]). The distribution of the solute concentration in the liquid phase is given by Equation (3).

(3) $$\left.{C\left(r\right)=C_{L}\left(T\right)+2\left(C\right.}_{0}-C_{L}\left(T\right)\right)\sum_{n=1}^{\infty }\frac{J_{1}\left(\gamma_{n}\right).e^{\frac{-\gamma_{n}^{2}}{a^{2}}Dt}}{\gamma_{n}\left\lceil J_{0}^{2}\left(\gamma_{n}\right)+J_{1}^{2}\left(\gamma_{n}\right)\right\rceil }.J_{0}\left(\gamma_{n}\frac{r}{a}\right),$$

(4) $$\frac{K}{D}\cdot a\cdot J_{0}\left(\gamma \right)=\gamma \cdot J_{1}\left(\gamma \right)$$

where $J_{0}$ and $J_{1}$ are the Bessel functions, and $\gamma_{n}$ are the roots of the Equation (4) of the order *n*. Additionally, the diffusive flow at the interface is also proportional to the changing radius of the liquid domain.

(5) $$\frac{\partial a}{\partial t}=-D\left(\frac{\partial C}{dr}\right),$$

Using the Equations (2)–(4), the time-dependent evolution of the radius of the liquid phase is formulated.

(6) $$\frac{da}{dt}=2D\left[{C_{L}\left(T\right)-C}_{0}\right]\sum_{n=1}^{\infty }\frac{{\left(\frac{K}{D}\cdot a\right)}^{2}\cdot e^{\frac{-\gamma_{n}^{2}}{a^{2}}Dt}}{a\left[\gamma_{n}^{2}+{\left(\frac{K}{D}\cdot a\right)}^{2}\right]},$$

The model allows for the extraction of the fraction of solid, which can later be compared with other models. Based on the parameter defining the behavior of this analytical solution, it can be limited to two analytical forms, each describing a different scenario (Appendix A). In the first case, solidification is dominated by the diffusion of the solute element in the liquid. It assumes slow heating and cooling close to equilibrium conditions.

(7) $$\frac{K}{D}\cdot a\gg 1;\;a=a_{0}-\gamma_{1}\sqrt{\frac{D*t}{16}},$$

In the second case, solidification is independent of the diffusion process and is instead dominated by the reaction kinetics at the interface. This condition resembles the rapid cooling involved in AM processes.

(8) $$\frac{K}{D}\cdot a\ll 1;\;a={\left(2CK^{2}t^{2}+{a_{0}}^{2}\right)}^{\frac{1}{2}}-CKt,$$

For this work, both limiting cases (diffusion-dominant and kinetics-dominant) will be considered and further compared to the correspondent experimental data. The values of the parameters used in our model are given in Table 1.

The characteristic value of the kinetic constant was taken from the evaluation of the typical time of coexistence of the two-phase (solid–liquid) zone. This can be evaluated from a known temperature gradient in the additive process and the scanning speed. Assuming the full dissolution of the liquid phase, we can take the left side of Equation (8) to be equal to zero and obtain an unknown value for *K.*

## 3. Experimental Work

### 3.1. Material

The material used for the experiments is nickel superalloy powder, specifically AMDRY Rene 80 and commercially pure Ni powder, with a particle size distribution (d_50_ = 77 µm) suitable for the LMD process. The base plate used is 304 L stainless steel. Table 2 presents the experimental chemical composition of both Rene 80 and the Ni powder as measured by inductively coupled plasma optical emission spectroscopy (ICP-OES).

### 3.2. Laser Metal Deposition

Initially, single line scan LMD experiments were conducted to determine an optimal parameter set. Three parameter sets were selected to produce a thin wall consisting of 20 layers, as shown in Table 3. The thin wall was approximately 60 mm long; each layer measured about 400–500 µm thick. A bidirectional laser scanning strategy was used.

**Figure 2:**
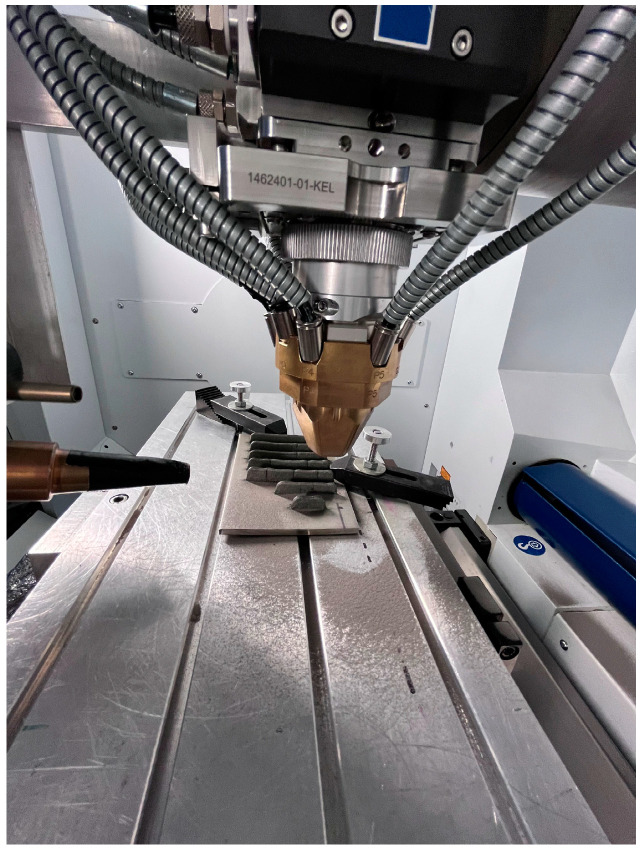
Experimental setup.

For the LMD experiments, a five-axis TruLaser Cell 3000 (TRUMPF Laser-un Systemtechnik SE, Ditzingen, Germany) working station, equipped with a 16 kW TruDisc 16002 (TRUMPF Laser-un Systemtechnik SE) Yb:YAG-disc laser with the wave length of 1030 nm, was used. The powder was deposited with a Medicoat Flowmotion Twin powder feeder. The working distance of the coaxial six-jet nozzle was 25 mm with a powder spot diameter of approx. 2.8 mm. The experimental setup is shown in Figure 2.

We used the following rules for the specimens’ codification: RE stands for Rene 80 alloy; NI stands for pure nickel specimens; the first two digits mean the scanning speed in m/min (i.e., 06 corresponds to 0.6 m/min); and the last two digits represent the number of the parameter set.

### 3.3. Temperature Measurement for LMD

Thermal cycles were measured in LMD experiments by means of a two-color pyrometer (Sensortherm Metis H322, Sensortherm GmbH, Steinbach, Germany). The thermal cycles’ spot position was located 5 mm above the base plate and 22 mm aside from the turn point of the LMD tracks. The measurements were repeated for pure nickel powder as a feedstock material as a reference. It is known that Ni has a much higher melting point correspondent to the considered Ni-based alloy, and this is why the cooling curve of Ni does not experience any kinks in the phase transition interval.

### 3.4. Differential Scanning Calorimetry (DSC)

Thermys One+(Setaram, Caluire, France) was used to perform thermos analytical measurements on the powder Rene 80 sample. The samples were heated in corundum crucibles (100 L) at a slow rate of 0.08 K/s (to replicate the equilibrium cooling condition) up to a maximum temperature of 1450 °C under argon (30 mL/min). The heating and cooling cycle was repeated after cooling to room temperature at a rate of 0.08 K/s. DSC curves provide information about the typical temperature at which significant phase transformation occurs, such as T_L_ and T_S_.

### 3.5. Thermodynamic Calculations

Calphad calculations were conducted using the TCHEA4 database through Thermo-Calc software (Thermo-Calc Software AB, Solna, Sweden). Scheil and equilibrium solidifications were calculated for the Rene 80 alloy using the experimentally determined composition. The Scheil–Gulliver equation defines the solute redistribution during the solidification of an alloy assuming perfect mixing in the liquid and no diffusion in the solid phase [20].

### 3.6. Microstructure Characterization

The metallographic cross-sections were cut out of the middle of an AM specimen and prepared by grinding them with sandpaper down to 1200 size, polishing with diamond suspension with 6 µm, 3 µm, and 1 µm grain size as well as 1/4 µm oxide particle suspension, and finally etching with Adler etchant. The DAS was calculated using image analysis software ImageJ 1.52.e (National Institutes of Health, Bethesda, MD, USA). SEM images were provided for the above prepared specimens by means of the scanning electron microscope LEO Gemini 1530VP (LEO Electron Microscopy Inc., Thornwood, NY, USA). For EDS analysis, the XFlash Detector 5030 (Bruker, Billerica, MA, USA) was used. For the elemental analysis, the ESPRIT 1.9 software of the same company was used.

## 4. Results and Discussion

### 4.1. Thermo-Calc Calculations

**Figure 3:**
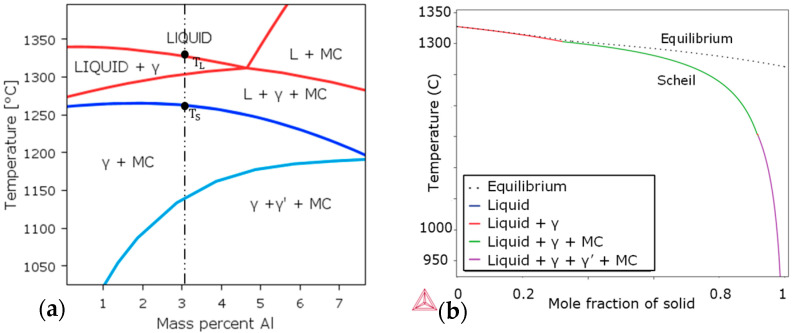
(**a**) Equilibrium phase diagram of Rene 80; (**b**) Scheil calculations of Rene 80.

The equilibrium phase diagram of the Rene 80 alloy, calculated using Thermo-Calc, is presented in Figure 3a. Under equilibrium conditions, the solidified material is expected to contain γ, γ′, and MC-type carbides. The solidification range, which represents the temperature range over which solidification occurs, is approximately 67 °C in equilibrium conditions. However, in non-equilibrium conditions, the solidification range increases to around 400 °C, which is six times higher than in the equilibrium state. This indicates that solidification takes place over a wider temperature range under non-equilibrium conditions.

In Figure 3b, the fraction of the dependences of the solid phases on the temperature during solidification is shown for Rene 80 based on the experimentally measured composition in Table 1. The Scheil model is calculated based on the local equilibrium existing between the solid and the liquid at the interface. No back diffusion of the solute is considered. Since the liquid enriched with the solute reaches the eutectic composition, the liquid phase can exist at a much lower temperature than in the equilibrium condition (represented by dotted lines).

### 4.2. Differential Scanning Calorimetry Analysis

**Figure 4:**
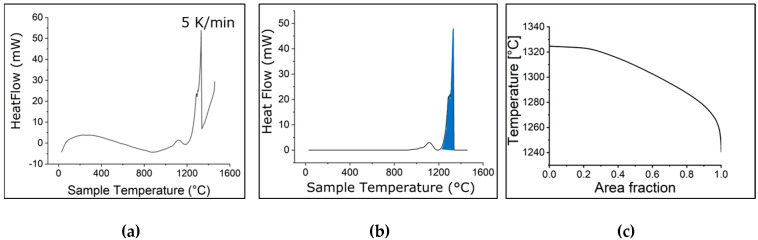
(**a**) DSC signal of Rene 80 as a function of temperature; (**b**) baseline subtraction; (**c**) the calculated area of solidified fraction.

The DSC measurement results are presented in Figure 4, where the condition closely resembles equilibrium. Figure 4a displays the DSC curve, illustrating the exothermic reaction observed during solidification. The baseline is subtracted from the DSC curve, and the area of phase transformation is shaded, as shown in Figure 4b. The onset of solidification is observed at approximately 1328 °C, while completion occurs around 1242 °C. The area of total heat flow is quantified, and in Figure 4c, it is converted into the fraction of solidification to facilitate comparison with other models. Notably, the solidification range obtained from the DSC curve is approximately 86 °C, slightly higher than predicted by Thermo-Calc for the equilibrium case.

### 4.3. LMD Thermal Cycles and Differential Thermal Analysis (DTA)

**Figure 5:**
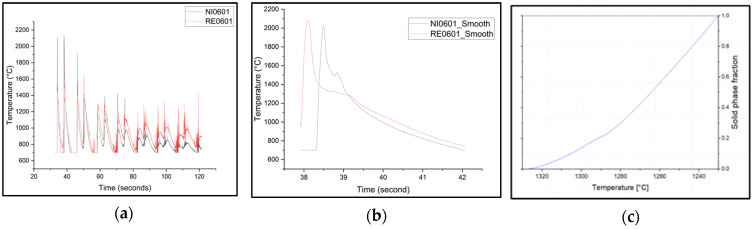
(**a**) Experimental thermal curve of Rene 80 specimen (RE0601) and pure nickel (NI601); (**b**) 2nd peak selected in both curves (smoothened using adjacent values); (**c**) the calculated area of solidified fraction.

The method of differential thermal analysis (DTA) has been applied to obtain the temperature-dependent solid fraction in LMD-manufactured specimens based on the measured cooling curves. The procedure resembles the previously described DSC, but in this case, we evaluated the first derivative of the cooling curves, which is proportional to the heat flux. As a reference, we have used the cooling curve of pure nickel, which represents only the heat flux associated with heat conduction. In a similar manner to that described above, the solid fraction can be estimated. An example of an evaluation is shown in Figure 5.

### 4.4. Microstructure Characterization

**Figure 6:**
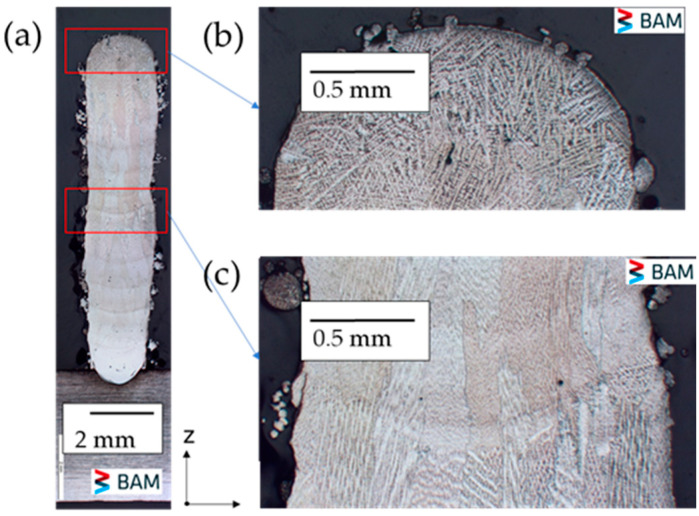
(**a**) Cross-sectional microstructure of the as-built thin wall, (**b**) fine dendrites in the top of the deposited layer, (**c**) columnar dendrities growing epitaxially from substrate.

Figure 6a depicts the cross-section of the as-built thin wall, providing insight into its microstructure. The topmost layer exhibits a fine dendritic structure (Figure 6b), while the middle layer demonstrates larger grains due to cyclic reheating and cooling effects. Epitaxial growth results in elongated columnar dendritic grains along the build direction (z) from the substrate (Figure 6c). This preferential growth is associated with the opposing heat flux from top to bottom and can be further observed in Figure 6.

**Figure 7:**
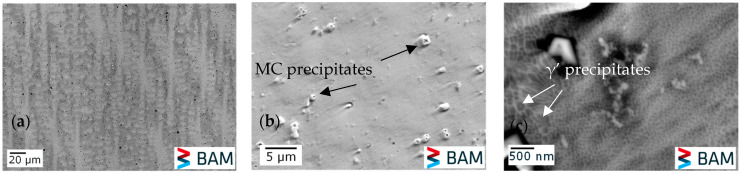
(**a**) Columnar dendritic grains; (**b**) MC precipitates; (**c**) γ′ precipitates.

Figure 7 displays the scanning electron microscope (SEM) images of the as-built thin wall. The DAS was calculated using image analysis software ImageJ, where a line was drawn across several dendritic arms and the arm spacing was determined by dividing the number of dendrites by the line length (Figure 7a). The average DAS was estimated to be 6.5 µm ± 0.5 µm, which was used as an input parameter (*a*_0_) in our model. Additionally, Figure 7b,c provide evidence of the presence of carbides and γ′ precipitates, respectively, supporting the Thermo-Calc findings. The size of the γ′ precipitates ranges between 20 nm and 100 nm. These findings correlate with those previously reported in [21].

**Figure 8:**
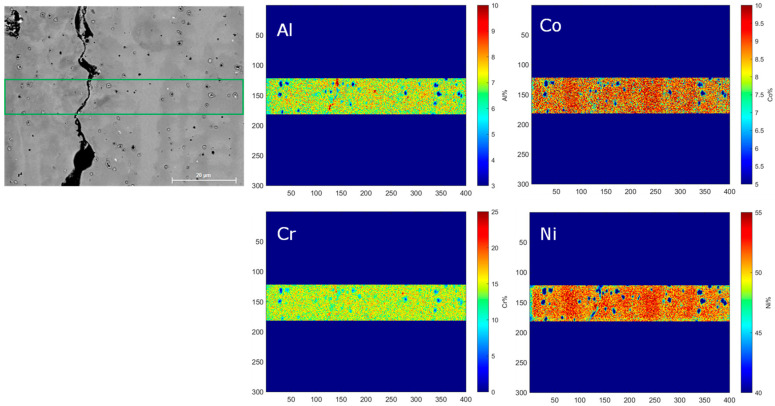
Elemental segregation of Al, Co, Cr, and Ni in columnar dendritic region.

**Figure 9:**
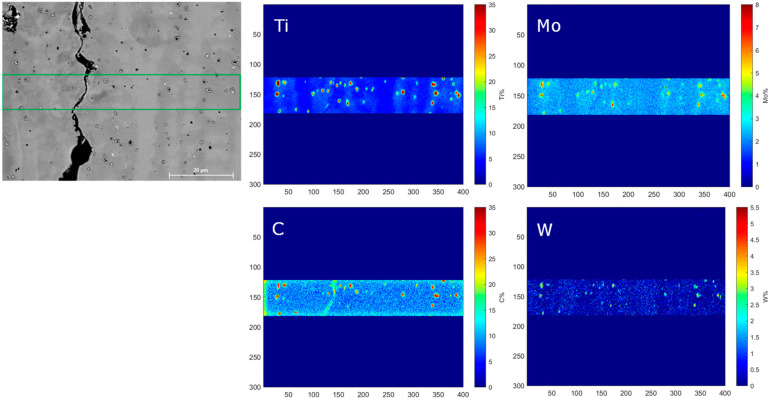
Elemental segregation of Ti, Mo, W, and C in interdendritic region.

The electron diffraction spectroscopy (EDS) mapping of a selected area of the Rene 80 samples shown in Figure 8 reveals the distinct segregation patterns of different elements. Specifically, Al, Co, Cr, and Ni are found to segregate predominantly within the columnar dendritic region, while Ti, Mo, W, and C tend to segregate in the interdendritic region, as shown in Figure 9.

This elemental segregation behavior can be related to the solidification microstructures forming in the Rene 80 alloy during the AM process. During solidification, the rapid cooling and solidification rates in the columnar dendritic region promote the formation of primary dendrites. The segregation of Al, Co, Cr, and Ni in this region can be attributed to their higher affinity for dendritic growth and their solubility in the γ phase of the alloy. On the other hand, the interdendritic region experiences slower cooling rates and provides more time for solute diffusion. Consequently, elements such as Ti, Mo, W, and C, which have lower solubility in the γ phase, tend to accumulate in the interdendritic regions due to their limited diffusion within the γ matrix and tend to promote MCs in the interdendritic region.

The observed elemental segregation patterns in the columnar dendritic and interdendritic regions are consistent with the known solidification behavior of alloy systems, as mentioned previously [3,4,5]. These results contribute to our understanding of the microstructural evolution and solidification mechanisms in the Rene 80 alloy during the AM process, which would help to extend our analytical model in the case of the consideration of secondary phases such as MCs and γ′, which are not currently covered by the model. This would help to optimize the manufacturing parameters and properties of the alloy in the future.

### 4.5. Validation of the Analytical Model

**Figure 10:**
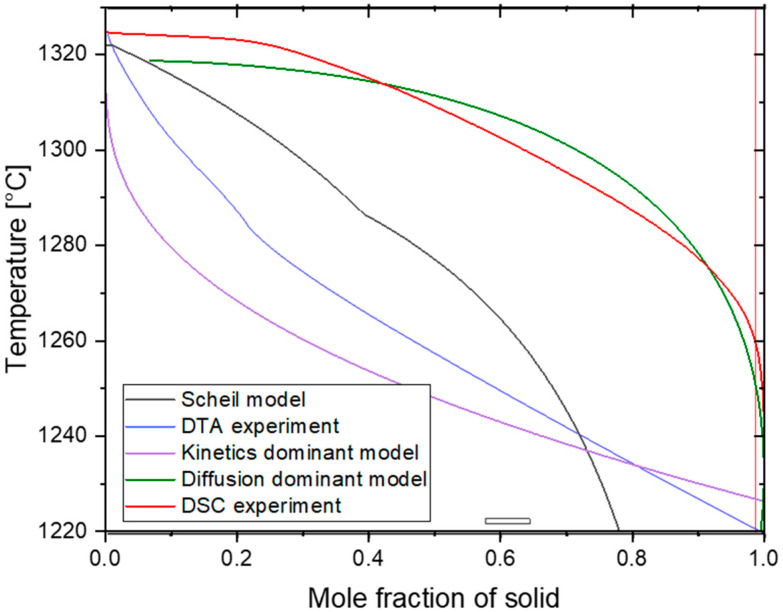
Validation of the analytical model with experimental data.

The comparison of the analytical model (diffusion-dominant) against other calculated models is presented in Figure 10. The DSC curve obtained at a cooling rate of 0.08 K/s represents the full equilibrium condition characterized by slow cooling. Comparing the diffusion-dominant analytical model (green line) with the DSC experiments (red line), a close agreement is observed between them. However, it is important to note that the Scheil curve, which considers only the local interface equilibrium, exhibits the presence of a liquid phase at significantly lower temperatures compared to the other curves.

While the agreement between the diffusion limiting analytical curve and the equilibrium curve provides confidence in the model’s performance under slow cooling conditions, it is essential to address the non-equilibrium solidification associated with rapid cooling during additive manufacturing. The comparison between the obtained experimental data (blue line) and the model curve in the case of kinetics-dominated solidification behavior (lilac line) shows that the tendency of a much higher rate of phase transformation at the beginning (high temperatures) is characteristic for both cases in contrast to the equilibrium close phase transformation. This slows down at the end of the solidification again in contrast to the equilibrium case. Thus, the presented model covers both cases of slow and fast kinetics and includes the methodological description of non-equilibrium solidification, especially for AM conditions, which has never been reported before. The main advantage of this mathematical model compared to the well-established numerical methods like CA and PF simulation is the possibility of providing the comparatively fast analysis of constituent phase distribution in the course of solidification depending on the complex thermal cycle including the forward and reverse transformation of the considered phases as an effect of multiple reheating and cooling. At the same time, this method has no limitations of the well-known Scheil–Gulliver theory, assuming equilibrium at the solid–liquid interface and no back diffusion in solid. By extending the analytical model to consider non-equilibrium solidification (Equation (7)), where diffusion is negligible and interface kinetics dominate, the model can capture the complex dynamics of multiple thermal cycles and concurrent phase transformations (liquid—γ + γ′ + MC).

## 5. Conclusions

In this work, an analytical model is proposed to describe the solidification behavior of the Rene 80 alloy in AM process. The proposed model addresses the limitations of the existing Scheil model and focuses on the primary phase formation at the solid–liquid interface. The main advantage of the presented model is that no assumptions are made about the thermodynamic equilibrium conditions at the solidification front. Thus, the model is able to describe any cooling condition taking place during the solidification process. A further advantage is a potential ability to consider the direct and reverse reaction at the phase boundary, i.e., the capability to represent complex thermal conditions taking place during the AM process. To validate our model, we conducted Thermo-Calc calculations, which revealed the presence of γ, γ′, and MC phases. Microstructural examination confirmed these findings, highlighting the segregation patterns of elements in both the columnar dendritic and interdendritic regions. These observations provide valuable insights into the microstructural evolution of the Rene 80 alloy during the AM process and shed light on the underlying solidification mechanisms. Model validation was performed by comparing our diffusion-dominant analytical model with the DSC model and equilibrium model. Besides this, the model was also validated for the conditions of a real AM process, characterized by rapid cooling conditions. In both cases, the model showed better performance than conventional established models, i.e., the thermodynamic equilibrium and Scheil–Gulliver models, by comparing with correspondent experimental data. Remarkably, the results demonstrated a good agreement among these models. However, it is important to note that the non-equilibrium solidification model, which occurs during rapid cooling in the AM process, needs to be further improved to take into account the coexistence of several phases within the solidification range. Incorporating non-equilibrium solidification and capturing interface kinetics would significantly enhance the accuracy and applicability of our model in advanced manufacturing processes like AM.

## Figures and Tables

**Table 1:** Model parameters.

Parameter	Value
Diffusion coefficient, *D*	5 × 10^−5^ cm^2^/s
Kinetic constant, *K*	0.026 cm/s
DAS, 2 *a*	6.5 µm
Initial concentration, *C*_0_	0.05

**Table 2:** Chemical composition of Rene 80 and Ni powder.

Material	Cr	Co	Ti	W	Mo	Al	Ni
Rene 80 in wt.-%	14.2	9.6	5.1	4.1	4.1	3	Bal.
Pure Ni in wt.-%	-	-	-	-	-	-	99.8

**Table 3:** Parameter set used for the LMD experiments.

Number	Power P (W)	Scan Speed v (mm/min)	Feed Rate m (g/min)	Spot Diameter s (mm)
RE0601	800	600	15	1.6
NI0601	800	600	15	1.6
RE0602	1000	600	15	1.6
RE0809	800	800	21	2.0

## Data Availability

The raw data supporting the conclusions of this article will be made available by the authors on request.

## Appendix A

The differential equation for the evolution of the radius of the liquid phase (6) can be considered for two extreme cases. For the limiting case of a large value of $\;\frac{K}{D}.a\gg 1$, Equation (6) will take the form of

(A1) $$\frac{da}{dt}=2D\left[{C_{L}\left(T\right)-C}_{0}\;\right]\sum_{n=1}^{\infty }\frac{e^{\frac{-\gamma_{n}^{2}}{a^{2}}Dt}}{a}$$

In the limit of large values of $\Lambda =\frac{K}{D}.a$, the roots of Equation (4) tend to their limit values so that the solution for the equation of dendritic cell radius evolution does not depend on the coefficient *K*. In this case, the growth of the dendritic cell corresponds to the equilibrium state defined by the position of the equilibrium solubility curve *C_L_*(*T*). This equation can be integrated numerically, considering that the initial size of the liquid interlayer (double radius) is equal to the average dendrite spacing distance.

Alternatively, we can search for the solution to the corresponding diffusion equation as a sum of the solutions for equations of the form

(A2) $$\frac{da}{dt}=\frac{C}{2}\frac{e^{\frac{-A}{a^{2}}t}}{a}$$

where $C=-4D\left[C_{0}-C_{L}\left(T\right)\right];\;A={\gamma_{n}}^{2}D$. By substituting variables $x=a^{2}$, we come to

(A3) $$\frac{dx}{dt}=\text{C}e^{\frac{-A}{x}t}$$

Logarithmizing both parts, we obtain in explicit form *t*:

(A4) $$t=-\frac{x}{A}\;\ln\left(\frac{\dot{x}}{C}\right)$$

The parameter $p=\frac{dt}{dx}$ is introduced; by differentiating the expression (A4) for *t*, we obtain the following:

(A5) $$dt=\frac{dx}{A}\ln\left(pC\right)+\frac{x}{A}\frac{dp}{p}=pdx$$

or

(A6) $$dx\left(p-\frac{1}{A}\ln\left(pC\right)\right)=\frac{x}{A}\frac{dp}{p}$$

Both parts are divided by *x* ($p-\frac{1}{A}\ln\left(pC\right)$):

(A7) $$\frac{dx}{x}=\frac{dp}{Ap^{2}-p\ln\left(pC\right)}$$

After the integration and back substitution of $x=a^{2}$, we obtain the partial solution of interest from the system of equations:

(A8) $$\begin{array}{l} a=e^{\frac{1}{2}\int \frac{dp}{Ap^{2}-p\ln\left(pC\right)}}\\ t=\frac{a^{2}}{A}\;\ln\left(pC\right) \end{array}$$

To perform this, we take advantage of the small change in $\ln\left(pC\right)$ with respect to *p* and replace this function with a constant $\ln\left(pC\right)=B$ (it can be shown that B≈16) under the integral sign, which finally yields the following:

(A9) $$a\left(t\right)=a_{0}-\gamma_{1}\sqrt{\frac{D*t}{16}}$$

Consider now the opposite limiting case, where $\frac{K}{D}.a\ll 1$**.**

(A10) $$\frac{da}{dt}=2\frac{K^{2}}{D}\left[{C_{L}\left(T\right)-C}_{0}\;\right]\;a\sum_{n=1}^{\infty }\frac{e^{\frac{-\gamma_{n}^{2}}{a^{2}}Dt}}{\gamma_{n}^{2}}$$

Tabulated roots of Equation (4) [22] (p. 493) show that all terms of the series are negligibly small with respect to the first term, provided that $\frac{K\left(T\right)}{D}.a\ll 1$. Thus, given the smallness of $\gamma_{1}$, using the Taylor series expansion of the exponent e^−*x*^ ≅ (1 − *x*),

(A11) $$\;\frac{da}{dt}=2\frac{K^{2}}{D}\left[{C_{L}\left(T\right)-C}_{0}\;\right]\frac{1}{a}\;\left(\frac{a^{2}}{\gamma_{1}^{2}}-Dt\right)$$

For small values of Λ = $\frac{K}{D}.a$, the solution of Equation (4) has an asymptotic for $\gamma_{1}$ equal to $\gamma_{1}^{2}=2\Lambda$; then,

(A12) $$\frac{da}{dt}=CK\left(\frac{2K}{a}t-1\right)$$

where $C=\left[C_{0}-C_{L}\left(T\right)\right]$ is found as a sum of the complete solution of the homogeneous and partial solution of the inhomogeneous equations $a=a_{1}+a_{2}$.

Looking for a complete solution to the homogeneous equation

(A13) $$\frac{da}{dt}=C\left(\frac{2K^{2}}{a}t\right)$$

Integrating this equation, we obtain

(A14) $$a_{1}={\left(2CK^{2}t^{2}+{a_{0}}^{2}\right)}^{\frac{1}{2}}$$

The partial solution of the inhomogeneous equation can be found in the form

(A15) $$a_{2}=CKt$$

Then, the complete solution of the inhomogeneous equation is given in the following form:

(A16) $$a={\left(2CK^{2}t^{2}+{a_{0}}^{2}\right)}^{\frac{1}{2}}-CKt$$
